# Selected Impacts of Urban Heat Islands on Emergency Medical Services Utilization in Rhode Island

**DOI:** 10.5811/westjem.50699

**Published:** 2026-04-14

**Authors:** Katelyn Moretti, Yiwen Liang, John Matthew Nicklas, Baylor Fox-Kemper, Clara DiCerbo, Hamid Torabzadeh, Christopher H Schmid, Adam Aluisio

**Affiliations:** * Warren Alpert Medical School of Brown University, Department of Emergency Medicine, Providence, Rhode Island; † The University of Hong Kong, LKS Faculty of Medicine, School of Public Health, Hong Kong SAR, China; ‡ Brown University, Department of Earth, Environmental, and Planetary Sciences, Providence, Rhode Island; § Providence Emergency Management Agency, Providence, Rhode Island; || Brown University, Division of Biology and Medicine, Providence, Rhode Island; # Brown University School of Public Health, Department of Biostatistics, Providence, Rhode Island

## Abstract

**Introduction:**

Excessive environmental heat exposure is clearly associated with an increased likelihood that individual patients will suffer adverse health outcomes. Such heat exposure also strains healthcare systems via increased utilization, a burden which can challenge systems’ capacities. Health impacts vary geographically with urban heat islands potentially contributing to higher temperatures and greater health risks. However, those most vulnerable to this exposure are not well identified. Our objective in this novel study was to compare and quantify differences in emergency medical services (EMS) use by selected patients during hot days in Rhode Island. Patients were recruited from low socioeconomic residential locations, stratified by whether they accessed EMS from within one of the state’s “urban heat islands,” or from other locations without “heat island” effects. We also compared selected patient demographic characteristics, and other EMS run data, between events associated with EMS access from these two types of areas.

**Methods:**

This retrospective, cross-sectional cohort study evaluated how the probability of an EMS encounter varied in response to daily mean temperature and the urban heat island status of the encounter location. We aggregated EMS dispatch data, daily mean temperature, urban heat island classification and the Area Deprivation Index of the encounter location. A quasi-Poisson regression model assessed the relationship between EMS encounter frequency and potential risk factors including daily temperature, urban heat island status, year, day of the week, sex, age, and relevant interaction terms. The model was restricted to low socioeconomic, residential encounter locations to reduce confounding (noted elsewhere by year) and focus on the target population. The primary outcome was the rate ratio (RR) of EMS encounters for urban heat island locations vs locations without an urban heat island effect, in response to summer temperatures. Secondary outcomes included RRs of EMS encounters stratified by age, sex, weekday vs weekend, and year.

**Results:**

Higher temperatures were associated with increased EMS call rates across all demographic subgroups. A 5 °F (2.8 °C) increase in mean daily temperature was associated with an increase in an overall EMS encounter rate of 1.5% (RR, 1.015; 95% CI, 1.005–1.031, *P* = .004). On a weekday in 2021, at 75 °F degrees, 68 EMS encounters would be predicted for the residential, low socioeconomic status locations in the state while at 95 °F, 73 EMS encounters would be expected. The EMS rates were consistently higher in urban heat islands across all study years, after accounting for daily temperature, year, day of the week, demographic characteristics, population size and interactions between age, sex, urban heat island and weekday vs weekend. The largest relative increase in EMS encounters was observed in 2019, with rates 34% higher in urban heat islands compared to locations without an urban heat island effect (RR, 1.34; 95% CI, 1.27–1.42). The smallest increase occurred in 2020 (RR, 1.12; 95% CI, 1.06–1.18).

**Conclusion:**

In residential and low socioeconomic locations, living in an urban heat island increased the probability of an EMS encounter, highlighting potential compounding effects of social and environmental vulnerability. As climate change intensifies extreme heat events, locationally targeted interventions may be critical in reducing heat-related health impacts.

## INTRODUCTION

Hot weather negatively impacts humans through a variety of pathophysiological mechanisms, but not all are affected equally.^1^–^5^ First, the adverse effects of heat on health are not geographically uniform. Urban heat islands (UHI) occur because built environments (eg, parking lots, large buildings) result in hotter than average surface temperatures and contribute to intra-city temperature differences.^6^–^10^ People living in UHIs are especially susceptible to heat and heatwaves with increased hospitalizations and mortality.^11^–^15^ In Boston, Massachusetts, streets with a higher land surface temperature were more likely than cooler streets to be the sites of medical emergencies during heat advisories relative to the rate of medical emergencies during non-heat advisory periods.^16^

Second, patient characteristics increase risk. Older adults are particularly susceptible secondary to chronic health conditions, limited mobility, and social isolation.^17^–^19^ Individuals with lower socioeconomic status or marginalized populations face increased heat risks due to poor housing conditions, lack of air conditioning, and extended outdoor working hours. In over 96% of the largest United States (U.S.) urban areas, persons of color are more likely to live in neighborhoods with higher heat intensity than non-Hispanic White residents.^20^–^21^

Rhode Island is the fastest warming state in the contiguous U.S., having warmed 3.1 °F (1.7 °C) since 1900.^22^ These rising summer temperatures have been linked to more frequent emergency medical service (EMS) encounters^23^ and increased emergency department (ED) use in the state.^24^ However, prior studies have not accounted for the UHI effect or the effects of patient-level variables such as age or socioeconomic status.

In this study we assessed the impacts of summer temperature variability (between June and August of 2018–2021) on EMS utilization in Rhode Island by comparing UHI and non-UHI locations, while accounting for patient age and socioeconomic status. Understanding and anticipating fluctuations in EMS demand provides an important opportunity to strengthen preparedness and enhance climate-change adaptation within the emergency care system as summer temperatures rise. Moreover, quantifying the morbidity associated with UHIs can guide decision makers and urban planners in directing mitigation and cooling interventions toward the communities most affected.

## METHODS

### Location

Rhode Island is the smallest U.S. state by area, covering a little over 1,000 square miles, with a population of just over 1 million residents.^25^ The state includes a mix of dense urban centers, suburban neighborhoods, and rural areas. Most of the population resides in and around the Providence metropolitan area, where many UHIs exist.^26^

### Database Formation

We constructed an integrated dataset by merging six distinct data sources: EMS utilization records; daily temperature data; urban heat severity indices; socioeconomic status metrics; census demographics; and building type classifications. These were linked using geospatial and administrative identifiers such as date, address, 9-digit ZIP code, and the 12-digit Federal Information Processing Series (FIPS) code (Supplemental Table 1). Eligible encounters were those occurring between June and August of 2018–2021 and for which the recorded location could be successfully linked to an UHI index.

**Population Health Research Capsule**

What do we already know about this issue?

*Hot weather increases illness and emergency care use, with urban heat islands (UHI) and social vulnerability amplifying heat-related hospitalizations*.

What was the research question?

*How do UHIs affect summer emergency medical services (EMS) use in low socioeconomic status residential areas of Rhode Island?*

What was the major finding of the study?

*EMS use was up to 34% higher in UHI than in non-UHI locations (RR, 1.34; 95% CI, 1.27–1.42)*.

How does this improve population health?

*By identifying heat-vulnerable communities, these findings support targeted interventions to reduce EMS demand and heat-related illness as temperatures rise*.

#### EMS Utilization

We obtained EMS data from the Rhode Island National Emergency Medical Services Information System (NEMSIS) v3, maintained by the Center for Emergency Medical Services within the Rhode Island Department of Health. This dataset contains patient-level records of EMS encounters, including demographics and location of EMS contact.

#### Temperature Data

Daily minimum, maximum, and mean temperatures were retrieved from the National Centers for Environmental Information of the National Oceanic and Atmospheric Administration (NOAA), using readings from the weather station at Theodore Francis Green State Airport (now Rhode Island TF Green International) in Warwick, Rhode Island.

#### Urban Heat Island Severity

We measured UHI severity using raster data (grid-based spatial datasets) from the Trust for Public Land’s ParkServe map. This dataset, with a 30-meter spatial resolution, was developed from Landsat 8 Band 10 imagery obtained via the National Aeronautics and Space Administration and NOAA. We used Landsat data from 2018 were used to generate this raster map for 2018–2019 and applied updated 2020 Landsat data to 2020–2021. This dataset provided the urban heat severity relative to the surrounding area categorized on a scale from 0–5 using the Jenks Natural Breaks classification method.^27^ A value of 0 indicates a location at or below the mean city temperature (non-heat island), while a score of 5 represents severe heat exposure (8–12 °F or 4–7 °C above the mean). The temperature ranges associated with each index score are presented in Supplemental Table 2. The ParkServe UHI layer has been used in prior UHI analyses and cited in public-health and urban forestry studies as a standardized, nationally consistent urban heat indicator.^28^,^29^

#### Socioeconomic Status

We assessed socioeconomic status at the encounter location using the 2021 Area Deprivation Index (ADI v4.0.1), developed by the Health Resources and Services Administration.^30^ The ADI provides decile rankings (1–10) of census block groups, where 1 indicates the least deprived and 10 the most deprived, based on composite measures of income, education, employment, and housing quality.

#### Population Demographics

Age- and sex-specific population estimates for each census block group were obtained from the 2020 U.S. Census. These data were linked to EMS encounters using the 12-digit FIPS code.^31^

#### Residential Classification

**Figure 1:**
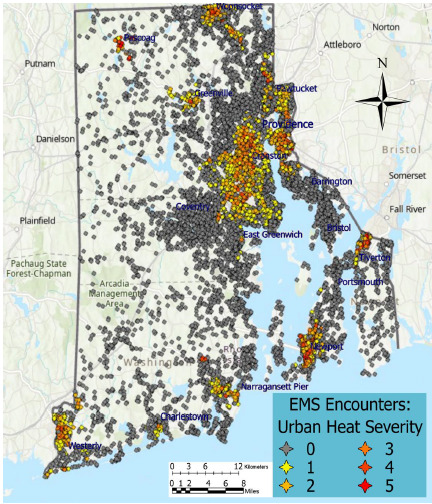
Locations in which emergency medical services were linked to urban heat severity classifications. Gray dots represent encounter locations without urban heat effect, Colored dots represent encounters categorized by urban heat index, ranging from 1 (yellow; lowest severity) to 5 (red; highest severity). *EMS*, emergency medical services.

Each encounter location was classified as residential (house, residential, duplex, apartment) or non-residential (commercial, industrial, government, educational) using data from the Rhode Island E-911 database.^32^ Linkage of the UHI classification to EMS encounter address via ArcGIS PRO (Environmental Research Services Institute, Redlands, CA)^33^ obtained an 85% success rate with only match scores > 40 accepted. Of all matches, 95% had a match score ≥ 80. Non-matches were all secondary to incomplete addresses. Non-matched encounters were manually reviewed, and the match rate was increased to 94% (Figure 1).

### Ethics Approval and Reporting Standards

We obtained institutional review board (IRB) approval from the Rhode Island Department of Health for access to patient-level data from the NEMSIS database (IRB 2020-08). Patient information was collected via standardized forms completed by EMS personnel for each encounter. All other datasets used in this analysis were publicly available and did not contain identifiable patient information. This study was conducted and reported in accordance with the Strengthening the Reporting of Observational Studies in Epidemiology guidelines.

### Variable Characterization

The final analytic dataset included the date of EMS encounter, patient demographics (age and sex), encounter location (including full address, 9-digit ZIP code, and FIPS code), UHI index at the encounter location, residential status, and the ADI score. To assess potential bias, we compared encounters excluded for insufficient data with the final analytic cohort using *t*-tests or chi-square tests.

The decision to dichotomize both UHI status (present vs absent) and ADI scores was based on both conceptual considerations and practical limitations identified during exploratory analyses. First, EMS encounters demonstrated a clear distributional shift at the boundary between urban heat severity = 0 and urban heat severity ≥ 1. In areas with an urban heat severity score of 0, most EMS runs originated from census block groups with ADI < 7, whereas in areas with any degree of urban heat severity (≥ 1), most encounters came from areas with ADI ≥ 7 (Supplemental Table 3). This suggested that the presence of any UHI exposure—not necessarily its graded severity—aligned with a meaningful sociodemographic shift in encounter patterns. In addition, the middle categories (ADI 4–6) did not form a stable or interpretable “medium” socioeconomic status group in our data; rather, they aligned more closely with the low-deprivation cluster (ADI 1–3).

Second, the frequency of EMS encounters within higher UHI categories was low. Only 15% of encounters originated from UHI levels ≥ 2, and UHI level 5 accounted for approximately one mean daily encounter. These sparse strata substantially limited statistical power and risked unstable estimates if modeled across the full five-level UHI scale as defined by the Trust for Public Land’s ParkServe map. Given these distributional constraints and the lack of sufficient events to support a multilevel approach, we selected dichotomization to preserve model stability and interpretability. Accordingly, we categorized UHI severity as non-UHI (urban heat severity = 0) and UHI present (urban heat severity ≥ 1) at the encounter location, with the latter encompassing all non-zero urban heat severity levels (Supplemental Table 2). Socioeconomic status was similarly dichotomized into high (ADI < 7) and low (ADI ≥ 7).

We dichotomized age as < 65 vs. ≥ 65 years, based on established evidence of increased heat vulnerability among older adults.^20^,^34^–^36^ Day of the week was classified as weekday vs weekend to account for known variations in EMS utilization.^36^,^37^ Encounter locations were classified as residential (e.g., house, duplex, apartment) or non-residential (e.g., commercial, industrial, government, educational settings). We compared cohort characteristics, including year, age group, and sex, stratified by urban heat severity and ADI scores, using Pearson’s chi-squared tests. Mean age and the proportion of residential encounters were also compared across these categories using *t*-tests.

### Model Development

We developed a quasi-Poisson regression model to determine the factors associated with the daily count of EMS encounters across Rhode Island. The model included patient-level covariates for age and sex (male vs female), and geographic covariate presence of an UHI at the encounter location. Temporal variables included year and day of the week, with the latter categorized as weekend vs weekday. Temperature exposure was modeled as the daily mean temperature, consistent with prior studies demonstrating comparable associations across mean, maximum, and minimum temperature metrics in Rhode Island.^23^ Lagged temperature exposures (up to five days) were evaluated but not found to be significant and were, therefore, excluded from the final model. We included a log-transformed population offset to account for varying population sizes across census blocks. The model evaluated all possible interactions. Those interactions found significant were included to assess potential effect modification across time and demographic subgroups.

Final analyses were restricted to encounter locations designated as residential and low socioeconomic status for several reasons. First, preliminary analyses revealed that confounding by year was present in non-residential, high socioeconomic status areas (Supplemental Figure 1). Second, individuals with high socioeconomic status are less vulnerable to environmental heat exposure.^17^–^18^ Additionally, it was reasoned that non-residential areas were most affected by COVID-19,^38^ did not represent prolonged exposures (eg, short errands), and were primarily characterized by indoor environments such as offices, airports, and shopping centers. Therefore, to reduce confounding and focus on the primary residential population of interest, we excluded EMS encounters from non-residential or high socioeconomic status locations.

To estimate the at-risk population living within UHIs in Rhode Island, we spatially joined 2020 census blocks with the 2021 UHI severity map. A census block was classified as located within an UHI if its centroid fell inside a designated heat island area. The populations of all low socioeconomic status blocks meeting UHI criteria were summed (Figure 3). Additionally, the population ≥ 65 of age within these blocks was aggregated by sex to enable age-sex stratified analysis.

## RESULTS

### Characteristics of Emergency Medical Services Encounters

**Figure 2:**
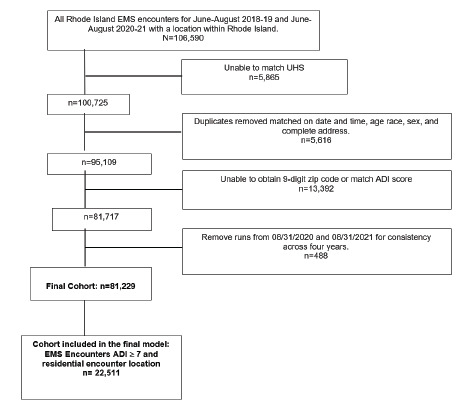
Cohort Selection Flow Diagram. Figure 2, This flow diagram depicts the cohort selection of EMS encounters included in the final analytic cohort, starting from 106,590 EMS encounters during summer the months (June–August) from 2018–2021, *ADI*, area deprivation index; *EMS*, emergency medical services; *SES*, socioeconomic status; *UHS*, urban heat severity.

A total of 106,590 EMS encounters were initially identified for the summer months (June–August) across 2018–2021. After removing duplicate records and restricting to encounters matched to a verifiable urban heat index, 95,109 encounters remained. We excluded an additional 13,392 encounters because of missing 9-digit ZIP codes and inability to determine an ADI score. To ensure temporal consistency across all years, we excluded August 31, missing from the 2018 and 2019 datasets, from the 2020 and 2021 datasets. The remaining number of EMS encounters was 81,229. For the Poisson regression model that was restricted to encounters in residential, low-SES encounter locations, the number of encounters was 22,511 (Figure 2).

We excluded a total of 13,880 encounters because they could not be linked to an ADI designation or occurred on the date of August 31 in 2020 or 2021. Compared with the included cohort, excluded encounters were less likely to originate from an UHI (55.2% vs 70.5%, *P* < .05), had a younger mean age (53 vs 58 years, *P* < .05), and had a higher proportion of males (50.8% vs 47.3%). There were no differences in the distribution of excluded encounters across study years (Supplemental Table 4).

The full summer temperature range across the four years of study was 42–100 °F (6–38 °C) with the mean daily temperatures covering a 52–87 °F (11–31 °C) range. The summer of 2019 was slightly cooler on average (72.2 °F, or 22.3 °C) and 2020 was slightly warmer (73.7 °F, or 23.1 °C) (Supplemental Figure 2). The mean daily number of EMS encounters during the study period was 223 (SD 23.6) (Supplemental Figure 2). For all EMS encounters including high socioeconomic status and non-residential locations, 55% originated from areas classified as non-UHIs and 59% occurred in higher socioeconomic status areas (computable from the header row of Table 1). Encounters in UHIs were more likely to be in low socioeconomic status areas (55%), and those without UHI effect more likely to be in high socioeconomic status areas (73%).

The overall mean patient age was 58 years (SD 23). Patients outside UHIs were older (mean 60 (SD 23]) than those from UHIs (mean 56 [SD 23]). Compared to patients from urban heat islands, those outside urban heat islands were more likely to be ≥ 65 years of age (49% vs 39%) and more likely to live in high socioeconomic status areas (73% vs. 42%).

A higher proportion of encounters not in UHIs occurred at residential locations (69%) vs. UHIs (65%; *P* < .001), and residential encounters were more common in low socioeconomic status areas (71%) than in high socioeconomic status areas (66%). Sex distribution was similar across strata, although male patients were slightly more represented in urban heat islands compared to locations without urban heat island effect (48% vs 47%).

### Baseline Population of Rhode Islanders Residing in an Urban Heat Island and low SES Status

**Figure 3:**
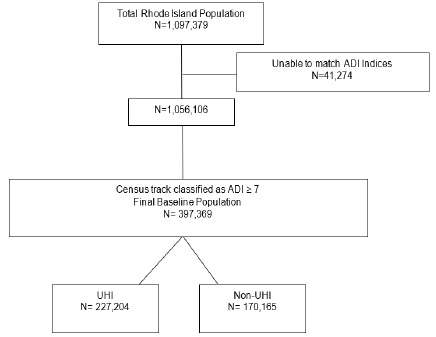
Baseline Populations in Rhode Island based on the 2020 census, the 2020 Trust for Public Land’s urban heat island severity layer and the 2021 Area Deprivation Index. *ADI*, Area Deprivation Index; *UHI*, urban heat island.

In 2020, the census of Rhode Island was 1,097,379. Several block groups did not have ADI values, either because the population was too small or the population lived in group quarters. This included local university campuses. These census blocks were excluded (total population excluded = 41,274). Among the remaining 1,056,106 residents, the number residing in a census track with an ADI ≥ 7 comprising the baseline population for regression was 397,369. Of those whose centroid overlapped with an UHI (urban heat severity ≥ 1 on the 2020 Trust for Public Land’s raster map) was calculated to be 227,2045 (Figure 3).

### Regression Analysis: EMS Encounters by Temperature, Urban Heat Island Effect, and Patient Characteristics for Low Socioeconomic, Residential Locations

Higher daily average temperatures increased the daily EMS call rates to residential, low-socioeconomic status locations and for all demographic subgroups. A 5 °F (2.8 °C) increase in daily mean temperature was associated with an increase in an overall EMS encounter rate of 1.5% (rate ratio [RR] 1.015, 95% CI, 1.005–1.031, P = .004, Supplemental Table 5 where RR is reported per 1 °F increase.) To illustrate the effect on the absolute total number of EMS responses, a weekday in 2021 was selected. From 75 °F to 95 °F, EMS encounters would be expected to rise from 68 EMS encounters to 73 EMS encounters.

**Figure 4:**
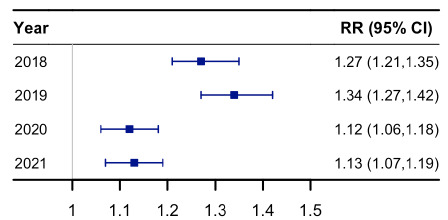
Adjusted rate ratios and 95% CI for EMS encounters among residents of urban heat island (UHI) areas compared with residents outside UHIs, shown by calendar year (2018–2021). Models were adjusted for daily temperature, year, day of the week, age, sex, population size, and interactions between age, sex, UHI status, and weekday vs weekend. Values > 1 indicate higher EMS encounter rates in UHI areas relative to non-UHI areas. *EMS*, emergency medical services *RR*, rate ratios; *UHI*, urban heat island.

The EMS encounter rates were consistently higher in areas classified as UHIs compared to areas outside UHIs across all study years, after accounting for daily temperature, year, day of the week, demographic characteristics, population size and interactions between age, sex, UHI, and weekday vs weekend. In 2018, EMS encounter rates in UHIs were **27% higher** than in areas without UHI effect (RR, 1.27; 95% CI, 1.21–1.35, Figure 4). This disparity increased in 2019, with a **34% higher** rate in UHIs (RR, 1.34; 95% CI, 1.27–1.42). Although the magnitude of association decreased slightly in subsequent years, UHIs continued to experience elevated EMS use in both 2020 (RR, 1.12; 95% CI, 1.06–1.18, Table 2) and 2021 (RR, 1.13; 95% CI, 1.07–1.19, Figure 4).

**Figure 5:**
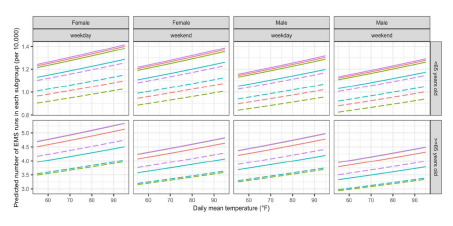
Associated number of EMS encounters per 10,000 people by daily mean temperature, stratified by age group (panel arranged horizontally), sex, and weekday vs weekend (panels arranged vertically). Estimates are derived from a quasi-Poisson regression of EMS encounters in Rhode Island for June–August 2018–2021. Lines represent associated EMS use across a temperature gradient (55 °F to 95 °F), stratified by year (color), urban heat island (UHI) status (solid: UHI, dashed: non-UHI), and demographic subgroup. Across all strata, EMS demand increased with temperature, with consistently higher associated rates in UHI areas. Trends were more pronounced in older adults (≥ 65 years) and during weekdays. *EMS*, emergency medical services; *UHI*, urban heat island.

Use of EMS increased substantially with higher temperatures, the presence of an UHI, and age (Figure 5). Utilization was slightly higher for females than for males, and slightly higher on weekdays than on weekends. For those < 65 of age living in a non-UHI, the associated number of EMS encounters per 10,000 people ranged between 0.8–1.1 at a temperature of 60 °F (16 °C), increasing to a range of 0.9–1.2 when the temperature reached 90 °F (32 °C). In an UHI, the rates were higher: approximately 1.1–1.2 on cool summer days and 1.3–1.4 on the warmest days. Conversely, the rates were much higher for individuals ≥ 65 years of age, ranging between 3.0–3.5 in non-UHI areas and 4.0–4.5 in UHI areas at 60 °F, to 3.0–4.5 in non-UHI areas and 4.5–5.5 in UHI areas at 90 °F.

These differences translated to a wide range of rate ratios (equivalent here to relative risk) comparing use rates at a fixed temperature for different combinations of sex, age, year, time of week and UHI residence to a reference group of males, < 65 years of age, on weekdays in non-UHI areas in 2020 (Table 2). Relative risks were all > 3 for older individuals and generally < 1.3 for those < 65 years of age. They were also lower in 2020 compared with other years in UHIs. In non-UHI areas, the rates were higher in 2020 than in 2018 or 2019 but lower than in 2021.

To illustrate the change in the absolute number of EMS encounters, we calculated the daily increase in encounters per 10,000 population (by subgroup) on weekdays in 2021, relative to a mean temperature of 75 °F, within residential, low socioeconomic locations. These values were then compared between areas with and without an UHI effect (Supplemental Figure 4). For example, among women > 65 years of age, an average daily temperature of 95° F (vs 75 °F) was associated with 0.3 additional EMS encounters per 10,000 people in low socioeconomic status residential locations without UHI, compared with 0.34 additional encounters in similar locations with UHI influence.

## DISCUSSION

Encounters with EMS increase as summer temperatures increase. This effect is found within all age and sex subgroups and across all days of the week. In addition, UHI exposure is associated with increased EMS utilization across Rhode Island, specifically in residential, low socioeconomic status areas. These findings underscore the disproportionate burden of heat-related health impacts among vulnerable populations and highlight the role of the local environment in healthcare utilization. Notably, EMS encounters were consistently higher in UHIs compared to non-UHIs, even after controlling for daily temperature and year. The strength of this association was most pronounced in 2018 and 2019, when the rate of calls was about 30% higher in heat-affected urban zones. This was despite a slightly cooler 2019 summer. Although the magnitude of the UHI effect diminished in 2020 and 2021, likely influenced by broader social disruptions and shifts in healthcare utilization related to the COVID-19 pandemic,^39^ the association remained statistically significant.

This aligns with prior research linking UHIs with higher rates of heat-related illness, including heat exhaustion and heat stroke.^40^ Other disease-specific analyses, such as cardiovascular hospitalizations, have also been linked to UHIs with a 2.4% increase observed during periods of extreme heat.^41^ Recent systematic review and meta-analysis further found a 6% higher risk of morbidity or mortality associated with UHIs during periods of elevated ambient temperatures. However, the review’s combined morbidity estimates drew from studies focused on specific diagnoses rather than overall illness burden. On the mitigation side, a natural cooling intervention in Canada, primarily consisting of expanded green space, was associated with a 40–50% reduction in heat-related ambulance calls.^42^ The current study adds to this evidence base by quantifying all-cause morbidity rather than limiting the analyses to specific diagnoses and by estimating the broader burden on the EMS system of an entire state.

Adaptive measures such as air conditioning (AC) have helped reduce the impact of extreme heat and have likely prevented some of the heat-related deaths that were previously projected.^43^–^44^ However, low socioeconomic status and income have been associated with limited capacity to cool built environments because of the high cost of electricity and the inability to build central cooling infrastructure.^45^ These vulnerable populations may instead have to rely on other interventions (ie, public buildings for AC, public pools, fans, increased hydration), which may not be feasible or effective in increasingly intense and frequent extreme heat events.^46^ As shown in the current analysis, EMS utilization was greater in UHIs in residential areas with lower socioeconomic status, and it further increased in response to rising summer temperatures. This clearly demonstrates the ongoing adverse health impacts of heat for vulnerable populations residing in urban heat islands.

## LIMITATIONS

While our analysis is based on complete, statewide EMS records and official National Weather Service temperature recordings over a four-year period, our data have some weaknesses. A subset of encounters (n = 13,880) could not be linked to an ADI designation or fell on August 31, 2020, or August 31, 2021 and were, therefore, excluded from the final cohort. Comparison of these excluded encounters with the included cohort revealed small but statistically significant differences between the groups. Excluded encounters were younger, less likely to originate from UHIs, and had a higher proportion of males. Although these differences were statistically significant, they appear to be of limited clinical significance. The excluded group represents a population somewhat less likely to experience increased EMS utilization with rising temperatures—given younger age, male predominance, and lower representation from UHIs. Even within the subset of excluded encounters originating from UHIs, the slightly younger age and higher proportion of males could contribute to a marginally lower temperature-related increase in EMS utilization, potentially yielding a slightly lower overall rate ratio. However, these differences were relatively small and would have been accounted for in age-sex stratified analyses.

Although our models adjusted for key demographic, temporal, and neighborhood-level factors, the possibility of residual confounding remains. Our Poisson models estimated average risk within age, sex, and ADI strata, rather than at the individual level. Because individual characteristics were not available for residents who did not use the EMS system, we were unable to account for person-level differences that may influence heat-related risk. As a result, residual confounding from unmeasured individual factors remains possible. Additional individual-level characteristics such as comorbidities, housing conditions, or access to AC, may also have influenced both heat exposure and EMS utilization and, therefore, resulted in confounding. By limiting the final analysis to lower socioeconomic status groups, variation in AC access is likely more comparable between UHI and non-UHI areas, as AC availability has previously been linked to socioeconomic status.^47^ In addition, age-stratified analyses help reduce potential confounding from comorbidities, which are strongly associated with age. These unmeasured factors may have partially contributed to the observed associations; however, they are unlikely to fully explain the consistent patterns seen across years, temperature ranges, and demographic strata.

In addition, this analysis only included EMS encounters linked to residential locations; therefore, certain at-risk populations were not captured in our cohort. These groups may include individuals experiencing homelessness, outdoor workers, and others who spend substantial time outside the home. Future research should specifically examine these populations to better understand their heat-related risks and EMS use patterns.

Because Rhode Island has only one official weather station, we could apply only one daily average temperature measurement across the entire state. Temperature variations throughout the state are approximated each day by interpolated data products (e.g., DAYMET) and machine-learning models (e.g., XIS-Temperature).^48^–^49^ We plan to use these in future studies. Additionally, the assumed temperature variability of urban heat islands was calculated geographically based on historic time-averaged Landsat-8 data. Actual surface temperatures at the time of the encounter were not used, as raw surface temperature data were only available roughly once per 24 days.^48^ Other important weather attributes that potentially modulate heat exposure—humidity, direct solar radiation, precipitation, and wind—were also not captured.

The EMS encounters were analyzed at the census block group level, UHI affect, and ADI levels based on the location of the EMS encounter. However, this location may not accurately reflect where they were exposed to heat, introducing potential misclassification. Limiting analyses to residential locations likely reduced misclassification from short-duration exposures in public or commercial venues (eg, parking lots, traffic accidents) and excluded large heat island zones that are typically air-conditioned, such as malls, airports, and office complexes.

We defined UHI exposure using a 2019 and 2021 map and retrospectively applied it across corresponding study years (2018–2021). This assumes spatial stability in UHI intensity, which may not fully account for temporal changes in land use, vegetation, or surface reflectivity that could influence local heat patterns. Further, our findings may not be generalizable beyond Rhode Island given its unique geography, climate change profile, and urban design.

## CONCLUSION

This study demonstrates that both elevated daily temperatures and exposure to an urban heat island are associated with increased EMS use during summer months in Rhode Island, specifically within residential areas that are both low socioeconomic status and urban heat islands. As climate change accelerates and extreme heat events become more frequent, EMS systems will continue to encounter temperature-related increases in demand. In this study, differences in EMS use between urban heat islands and non-urban heat island areas were measurable, reflecting unequal heat-related health impacts across communities. Targeted adaptations—such as enhancing local cooling infrastructure, improving heat-risk communication, and supporting high-risk neighborhoods with higher burdens of exposure to these heat islands—could help mitigate the disproportionate impacts of heat on vulnerable populations.

## Supplementary Information



## Figures and Tables

**Table 1:** Demographic and encounter characteristics by urban heat island status and area deprivation index (< 7 or ≥ 7) for all socioeconomic, non-residential and residential encounters. All percentages are calculated relative to the column header.

	Overall (N = 81,229)	Non-UHI (n = 44,847)	UHI (n = 36,382)	P-value (UHI)	High SES (n = 48,171)	Low SES (n = 31,734)	SES Missing (n = 1,324)	P-value (SES)
**Year**				<.001				<.001
2018	21,066 (25.9%)	11,357 (25.3%)	9,709 (26.7%)		12,610 (26.2%)	8,063 (25.4%)	393 (29.7%)	
2019	21,273 (26.2%)	11,506 (25.6%)	9,767 (26.9%)		12,865 (26.7%)	8,006 (25.2%)	402 (30.3%)	
2020	18,312 (22.5%)	10,477 (23.4%)	7,835 (21.5%)		10,880 (22.6%)	7,186 (22.7%)	246 (18.6%)	
2021	20,578 (25.3%)	11,507 (25.7%)	9,071 (24.9%)		11,816 (24.5%)	8,479 (26.7%)	283 (21.4%)	
Age Mean (SD)	58.0 (23.3)	59.9 (23.4)	55.8 (22.9)	<.001	61.1 (23.4)	53.9 (22.3)	47.5 (21.4)	< .001
Age				<.001				< .001
<65 yrs	45,155 (55.6%)	22,853 (51.0%)	22,302 (61.3%)		23,321 (48.4%)	20,830 (65.6%)	1,004 (75.9%)	
≥65 yrs	36,018 (44.4%)	21,966 (49.0%)	14,052 (38.6%)		24,822 (51.5%)	10,877 (34.3%)	319 (24.1%)	
Missing	56 (<0.1%)	28 (<0.1%)	28 (0.1%)		28 (0.1%)	27 (0.1%)	1 (<0.1%)	
Sex				.002				.002
Female	42,712 (52.6%)	23,789 (53.0%)	18,923 (52.0%)		25,459 (52.9%)	16,704 (52.6%)	549 (41.5%)	
Male	38,460 (47.3%)	21,025 (46.9%)	17,435 (47.9%)		22,685 (47.1%)	15,002 (47.3%)	773 (58.4%)	
Missing	57 (0.1%)	33 (0.1%)	24 (0.1%)		27 (<0.1%)	28 (0.1%)	2 (0.1%)	
% Residential Location	67.3%	69.4%	64.7%	< .001	66.1%	70.9%	23.0%	< .001
SES				<.001				
High	**48,171 (59.3%)**	32,854 (73.3%)	15,317 (42.1%)					
Low	**31,734 (39.1%)**	11,575 (25.8%)	20,159 (55.4%)					
Missing	**1,324 (1.6%)**	418 (0.9%)	906 (2.5%)					
UHI								
UHI					15,317 (31.8%)	20,159 (63.5%)	906 (68.4%)	
Non-UHI					32,854 (68.2%)	11,575 (36.5%)	418 (31.6%)	

*SES*, socioeconomic status; *UHI*, urban heat island.

**Table 2:** Rate ratio for the overall association of emergency medical services encounters with risk factors relative to the reference group (male, age < 65, non-urban heat island [UHI], weekday, 2020): by year, as well as combinations of year, sex/age group, and UHI exposure, associated with the rate ratios of daily EMS encounters for a fixed temperature. Estimates reflect combined main and interaction effects. Rate ratios for UHI are bolded.

			2018	2019	2020	2021
*Male < 65*	Weekday	Non-UHI	0.96 (0.89, 1.02)	0.90 (0.84, 0.96)	**1.00**	1.09 (1.02, 1.17)
	Weekday	UHI	1.22 (1.15, 1.29)	1.20 (1.13, 1.28)	1.12 (1.06, 1.18)	1.23 (1.16, 1.31)
	Weekend	Non-UHI	0.94 (0.87, 1.01)	0.88 (0.81, 0.95)	0.98 (0.95, 1.01)	1.07 (0.99, 1.15)
	Weekend	UHI	1.19 (1.11, 1.28)	1.18 (1.10, 1.26)	1.10 (1.03, 1.17)	1.21 (1.12, 1.29)
*Male* ≥ *65*	Weekday	Non-UHI	3.50 (3.25, 3.78)	3.45 (3.20, 3.73)	3.50 (3.25, 3.78)	4.12 (3.83, 4.44)
	Weekday	UHI	4.47 (4.16, 4.80)	4.64 (4.32, 4.99)	3.92 (3.61, 4.26)	4.65 (4.33, 4.99)
	Weekend	Non-UHI	3.17 (2.93, 3.42)	3.12 (2.89, 3.38)	3.17 (2.97, 3.37)	3.73 (3.46, 4.02)
	Weekend	UHI	4.03 (3.74, 4.34)	4.19 (3.89, 4.52)	3.54 (3.25, 3.86)	4.20 (3.90, 4.52)
*Female < 65*	Weekday	Non-UHI	1.03 (0.95, 1.10)	0.96 (0.89, 1.04)	1.07 (1.05, 1.10)	1.17 (1.09, 1.26)
	Weekday	UHI	1.31 (1.22, 1.40)	1.29 (1.21, 1.38)	1.20 (1.13, 1.28)	1.32 (1.24, 1.41)
	Weekend	Non-UHI	1.01 (0.93, 1.09)	0.94 (0.87, 1.02)	1.05 (1.01, 1.10)	1.15 (1.06, 1.24)
	Weekend	UHI	1.28 (1.19, 1.38)	1.27 (1.17, 1.36)	1.18 (1.10, 1.26)	1.29 (1.20, 1.40)
*Female* ≥ *65*	Weekday	Non-UHI	3.76 (3.48, 4.07)	3.71 (3.43, 4.02)	3.76 (3.53, 4.02)	4.43 (4.11, 4.79)
	Weekday	UHI	4.80 (4.45, 5.17)	4.99 (4.62, 5.38)	4.21 (3.86, 4.60)	5.00 (4.64, 5.38)
	Weekend	Non-UHI	3.40 (3.14, 3.69)	3.35 (3.09, 3.64)	3.40 (3.18, 3.64)	4.00 (3.70, 4.33)
	Weekend	UHI	4.33 (4.01, 4.68)	4.50 (4.17, 4.87)	3.80 (3.48, 4.16)	4.51 (4.18, 4.87)

*UHI*, urban heat island.
