# Supplementary material for: Selected Impacts of Urban Heat Islands on Emergency Medical Services Utilization in Rhode Island
Source: West J Emerg Med. 2026 Apr 14;27(3):490–500. doi: 10.5811/westjem.50699 (PMC13246169; doi:10.5811/westjem.50699)
Supplement: Supplementary file 1 [file wjem-27-490-s001.docx]

**SUPPLEMENTAL FIGURES**

| **Database** | **Key Variables included for analysis** | **Merge Variable** |
| --- | --- | --- |
| Rhode Island National NEMSIS V3 dataset | EMS encounter records, patient demographics | Date, address, 9-digit zip code, FIPS code |
| NOAA/National Centers for Environmental Information | Daily mean temperature | Date |
| Trust for Public Land’s UHI Severity Layer 2018, 2020 | Urban heat island index | Address |
| Area Deprivation Index v4.0.1 (2021) | ADI score | 9-digit zip code |
| 2020 U.S. Census | Population by age and gender | FIPS code |
| Rhode Island E-911 | Residential vs. non-residential classification | Address |

Supplemental Table 1: The six databases merged with key variables utilized.

| Urban Heat Index | 0 | 1 | 2 | 3 | 4 | 5 |
| --- | --- | --- | --- | --- | --- | --- |
| Severity | None | Very Low | Low | Moderate | High | Very High |
| Temperature above mean (°F) | At or below mean | 1 | 2-3 | 4-5 | 6-7 | 8-12 |

Supplement Table 2: Degrees range for urban heat severity category definitions

|  | | UHI Index | | | | | | p-value |
| --- | --- | --- | --- | --- | --- | --- | --- | --- |
|  |  | 0 | 1 | 2 | 3 | 4 | 5 |  |
| Total EMS runs |  | 44,847 | 11,903 | 12,004 | 9,737 | 2,414 | 324 |  |
| Mean daily EMS runs |  | 123 | 33 | 33 | 27 | 7 | 1 |  |
| Year | 2018 | 11,357 (25%) | 3,025 (25%) | 3,237 (27%) | 2,798 (29%) | 582 (24%) | 67 (21%) | <0.001 |
|  | 2019 | 11,506 (26%) | 3,043 (26%) | 3,158 (26%) | 2,865 (29%) | 627 (26%) | 74 (23%) |  |
|  | 2020 | 10,477 (23%) | 2,759 (23%) | 2,515 (21%) | 1,944 (20%) | 535 (22%) | 82 (25%) |  |
|  | 2021 | 11,507 (26%) | 3,076 (26%) | 3,094 (26%) | 2,130 (22%) | 670 (28%) | 101 (31%) |  |
| Age (Mean (SD)) |  | 59.87 (23.40) | 57.34 (23.42) | 55.83 (23.15) | 54.92 (22.44) | 51.35 (20.47) | 57.66 (20.80) | <0.001 |
| Age Category | <5 | 492 (1.1%) | 145 (1.2%) | 140 (1.2%) | 98 (1.0%) | 25 (1.0%) | 1 (0.3%) | <0.001 |
|  | 5-65 | 22,361 (50%) | 6,687 (56%) | 7,144 (60%) | 6,125 (63%) | 1,750 (73%) | 187 (58%) |  |
|  | >65 | 21,966 (49%) | 5,059 (43%) | 4,712 (39%) | 3,507 (36%) | 638 (26%) | 136 (42%) |  |
| Gender | Female | 23,789 (53%) | 6,355 (53%) | 6,252 (52%) | 4,998 (51%) | 1,138 (47%) | 180 (56%) | <0.001 |
|  | Male | 21,025 (47%) | 5,537 (47%) | 5,747 (48%) | 4,735 (49%) | 1,272 (53%) | 144 (44%) |  |
|  | Unknown (Unable to determine) | 33 (<0.1%) | 11 (<0.1%) | 5 (<0.1%) | 4 (<0.1%) | 4 (<0.1%) | 0 (0%) |  |
| ADI | 1 | 5,084 (11%) | 335 (2.8%) | 319 (2.7%) | 471 (4.8%) | 146 (6.0%) | 5 (1.5%) | <0.001 |
|  | 2 | 6,171 (14%) | 386 (3.2%) | 292 (2.4%) | 397 (4.1%) | 392 (16%) | 22 (6.8%) |  |
|  | 3 | 6,222 (14%) | 1,102 (9.3%) | 605 (5.0%) | 387 (4.0%) | 130 (5.4%) | 46 (14%) |  |
|  | 4 | 6,152 (14%) | 1,344 (11%) | 1,033 (8.6%) | 1,147 (12%) | 145 (6.0%) | 10 (3.1%) |  |
|  | 5 | 3,772 (8.4%) | 1,068 (9.0%) | 1,500 (12%) | 974 (10%) | 291 (12%) | 51 (16%) |  |
|  | 6 | 5,453 (12%) | 976 (8.2%) | 990 (8.2%) | 570 (5.9%) | 173 (7.2%) | 10 (3.1%) |  |
|  | 7 | 3,515 (7.8%) | 1,706 (14%) | 1,644 (14%) | 1,209 (12%) | 256 (11%) | 25 (7.7%) |  |
|  | 8 | 2,950 (6.6%) | 1,608 (14%) | 1,689 (14%) | 1,240 (13%) | 125 (5.2%) | 36 (11%) |  |
|  | 9 | 2,533 (5.6%) | 1,638 (14%) | 1,567 (13%) | 1,104 (11%) | 148 (6.1%) | 1 (0.3%) |  |
|  | 10 | 2,577 (5.7%) | 1,624 (14%) | 2,105 (18%) | 1,772 (18%) | 546 (23%) | 116 (36%) |  |
|  | GQ^1^ | 359 (0.8%) | 116 (1.0%) | 256 (2.1%) | 308 (3.2%) | 60 (2.5%) | 1 (0.3%) |  |
|  | PH^2^ | 0 (0%) | 0 (0%) | 0 (0%) | 158 (1.6%) | 2 (<0.1%) | 1 (0.3%) |  |
|  | U^3^ | 59 (0.1%) | 0 (0%) | 4 (<0.1%) | 0 (0%) | 0 (0%) | 0 (0%) |  |

Supplemental Table 3: EMS response and patient characteristics from areas with different urban heat island (UHI) severity index in Rhode Island, June-August 2018-2021 (% within UHI level).

|  | **Excluded** | **Included** | **P Value** |
| --- | --- | --- | --- |
|  | 13,880 | 81,229 |  |
|  |  |  |  |
| **Mean Age (SD)** | 53 (23.4) | 58 (23.3) | <0.05 |
| **Sex (%,)** |  |  |  |
| Male | 7,051 (50.8) | 38,460 (47.3) | <0.05 |
| Female | 6,817 (49.2) | 42,712 (52.6) |  |
| **Year (%)** |  |  |  |
| 2018 | 3,498 (25.3) | 21,066 (25.9) | 0.2 |
| 2019 | 3,672 (26.5) | 21,273 (26.2) |  |
| 2020 | 3,072 (22.2) | 18,312 (22.5) |  |
| 2021 | 3,600 (26.0) | 20,578 (25.3) |  |

Supplemental Table 4: Characteristics of excluded encounters analyses compared to the included total cohort using t-tests for continuous variables and chi-square tests for categorical variables.

***Initial Exploratory Analysis****.*

The distribution of EMS encounters from areas with different UHI severity levels remained similar for 2018 and 2019, with a notable overall decrease in total EMS encounters in 2020. EMS encounters rebounded in 2021, with a higher number of calls from UHIs compared to 2018 and 2019 (Table 3 and Supplemental Table 2).

Year specific estimates of the number of EMS encounters per day by the proportion of EMS encounters from urban heat islands varied by more than 10% from the overall estimates. When examining residential, low SES locations only, confounding by year resolved (Supplemental Figure 3).

Given the vulnerability of this population, this was also identified as the primary population of interest.


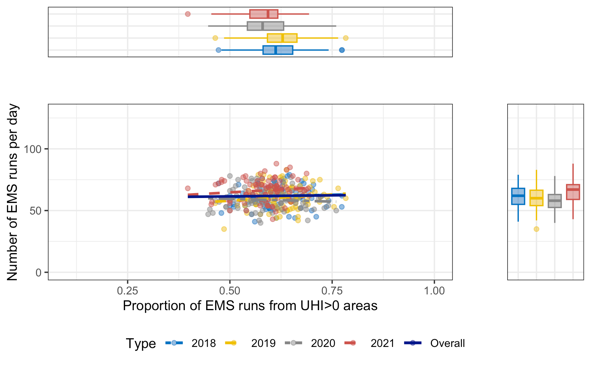

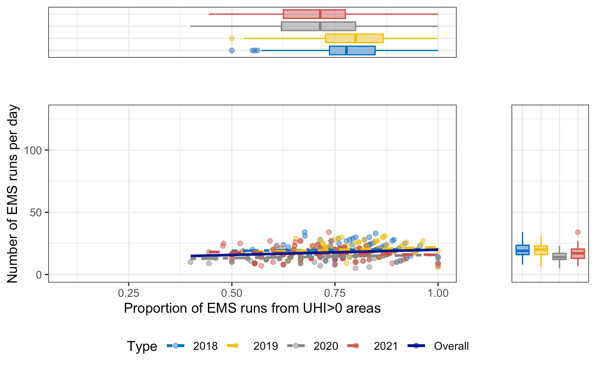


Figure 1(a) Residential, low socioeconomic. Figure 2(b) Non-residential, low socioeconomic.


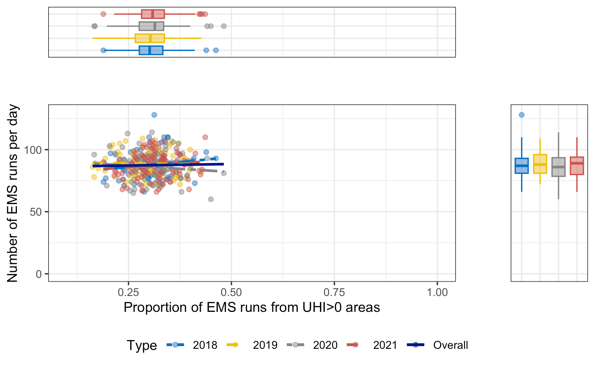

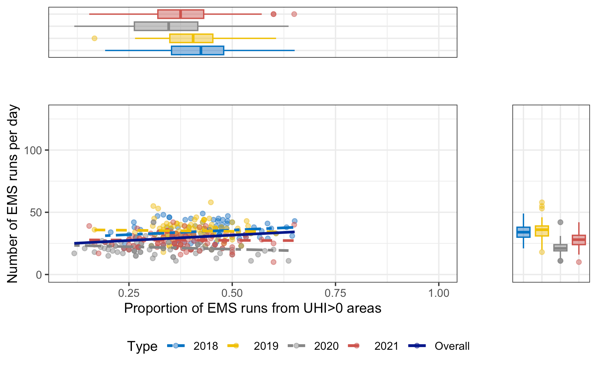


*Figure 3(c) Residential, high socioeconomic. Figure 4 (d) Non-residential, high socioeconomic.*

Supplemental Figure 1: Confounding illustration using linear association between daily EMS counts and proportion of runs from UHI>0 areas with boxplots representing marginal distributions in (a) residential, low socioeconomic areas, (b) non-residential, low socioeconomic areas, (c) residential, high socioeconomic areas, (d) non-residential, high socioeconomic areas.


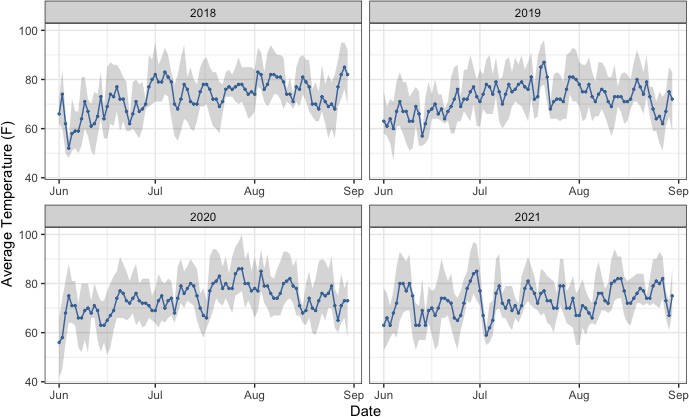
Supplemental Figure 2. maximum, minimum, and average daily temperatures (°F) from June to August for the years 2018 to 2021. The blue line represents the average temperature, while the shaded area is bounded by the minimum and maximum temperatures.


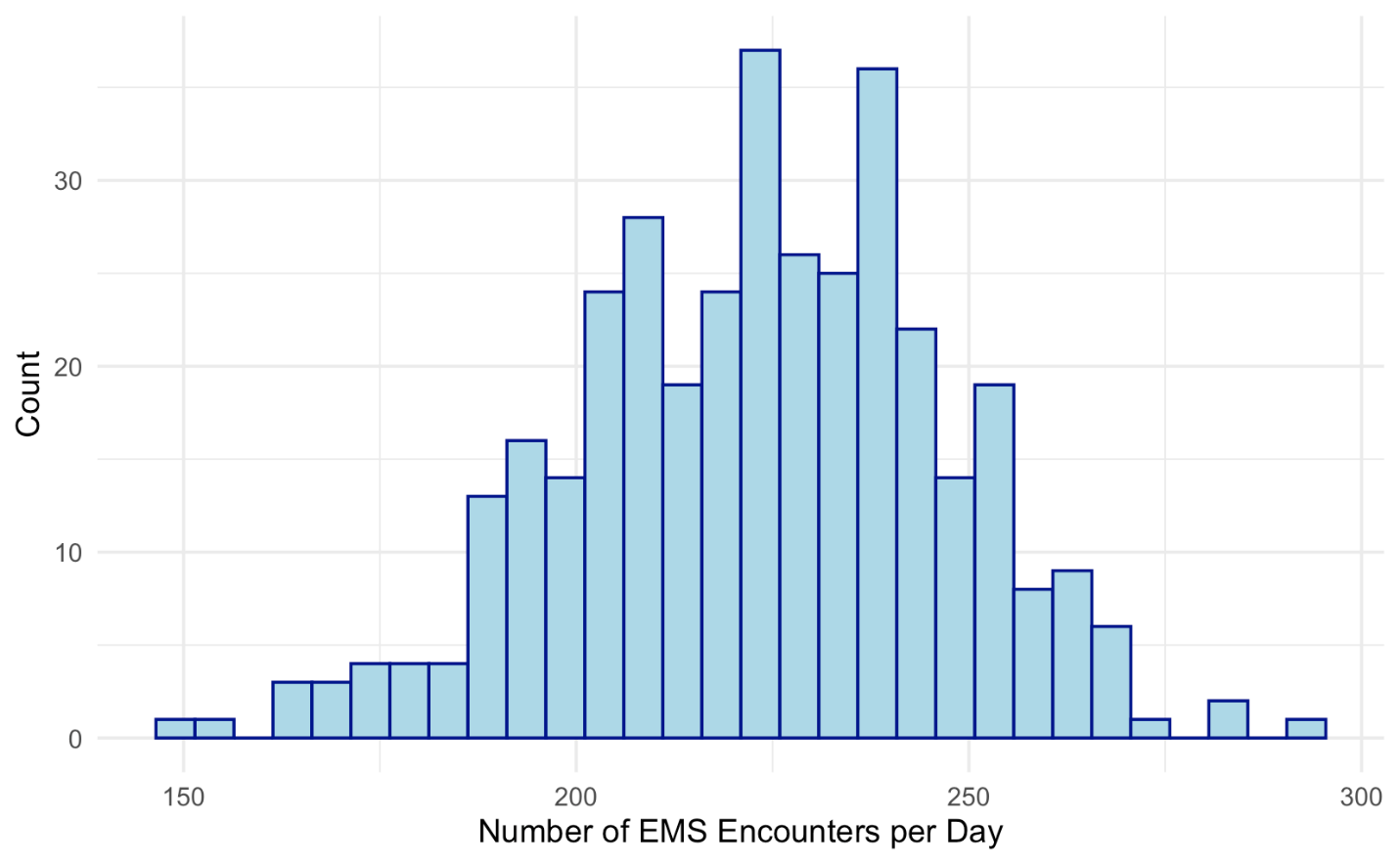


Supplement Figure 3: Distribution of the daily number of EMS encounters.

| **Variable** | **Rate Ratio (RR)** | **95% CI** | **p-value** |
| --- | --- | --- | --- |
| **Temperature** |  |  |  |
| Daily Average Temperature (°C) | 1.003 | (1.001, 1.006) | 0.004 |
| **Year (ref: 2020)** |  |  |  |
| 2018 | 0.96 | (0.89, 1.03) | 0.188 |
| 2019 | 0.90 | (0.84, 0.97) | 0.002 |
| 2021 | 1.09 | (1.02, 1.17) | 0.009 |
| Weekend | 0.98 | (0.95, 1.01) | 0.246 |
| Female | 1.07 | (1.05, 1.10) | <0.001 |
| **Age** ≥65 **(ref: <65)** | 3.50 | (3.30, 3.71) | <0.001 |
| **Urban Heat Island** | 1.12 | (1.06, 1.18) | <0.001 |
| **Interactions** |  |  |  |
| 2018 × UHI | 1.14 | (1.05, 1.23) | 0.001 |
| 2019 × UHI | 1.20 | (1.11, 1.30) | <0.001 |
| 2021 × UHI | 1.01 | (0.94, 1.09) | 0.852 |
| 2018 × Age ≥65 | 1.05 | (0.97, 1.15) | 0.254 |
| 2019 × Age ≥65 | 1.10 | (1.02, 1.21) | 0.015 |
| 2021 × Age ≥65 | 1.08 | (1.00, 1.18) | 0.051 |
| Weekend × Age ≥65 | 0.92 | (0.87, 0.97) | 0.004 |

Supplement Table 5: Rate ratios (RR) and 95% confidence intervals (CI) for the association between daily EMS encounters and mean daily temperature (°F), calendar year, day of the week, gender, age, and urban heat island exposure. The model includes an offset for log population size. Statistical significance was defined as *p* < 0.05.

***Initial Exploratory Analysis****.*

The distribution of EMS encounters from areas with different UHI severity levels remained similar for 2018 and 2019, with a notable overall decrease in total EMS encounters in 2020. EMS encounters rebounded in 2021, with a higher number of calls from UHIs compared to 2018 and 2019 (Table 3 and Supplemental Table 2).

Year specific estimates of the number of EMS encounters per day by the proportion of EMS encounters from urban heat islands varied by more than 10% from the overall estimates. When examining residential, low SES locations only, confounding by year resolved (Supplemental Figure 3).

Given the vulnerability of this population, this was also identified as the primary population of interest.


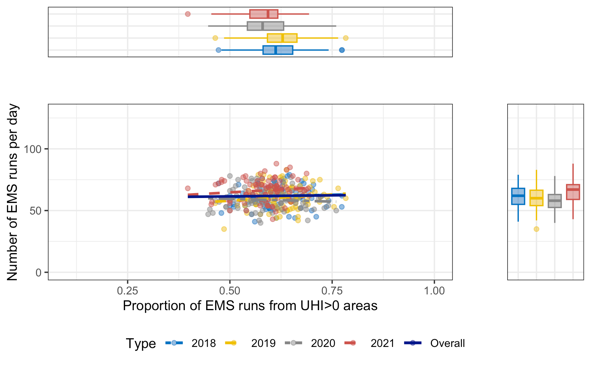

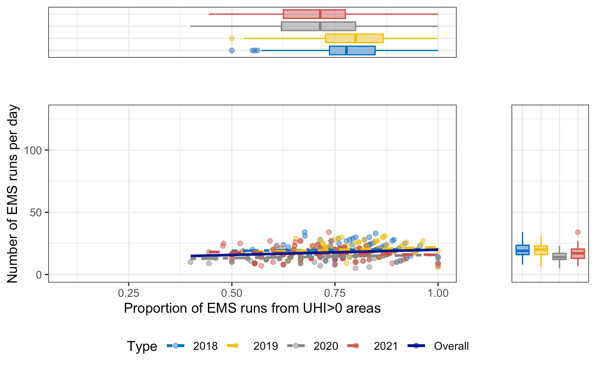


Figure 1(a) Residential, low socioeconomic. Figure 2(b) Non-residential, low socioeconomic.


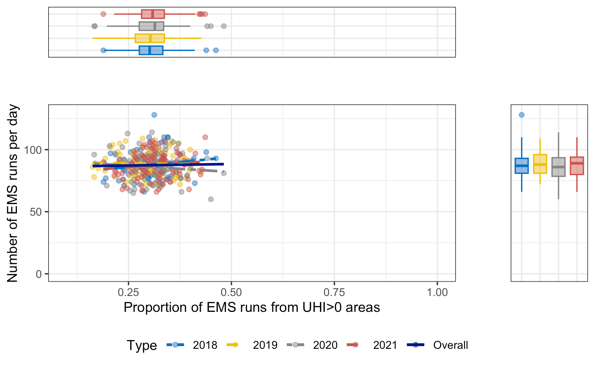

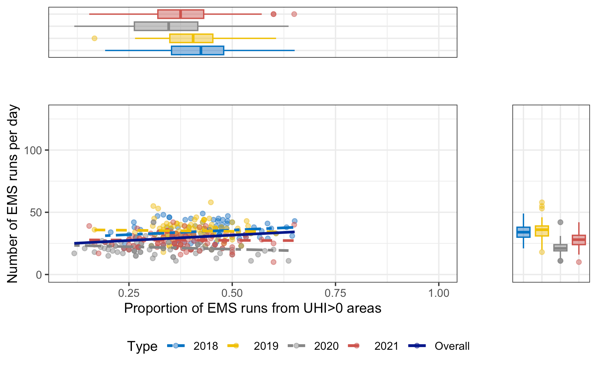


*Figure 3(c) Residential, high socioeconomic. Figure 4 (d) Non-residential, high socioeconomic.*

| **Panel A: No UHI Effect** | | | | | **Panel B: UHI Effect** | | | | |
| --- | --- | --- | --- | --- | --- | --- | --- | --- | --- |
| *Demographics* | *Daily Average Temperature (°F)* | | | | *Demographics* | *Daily Average Temperature (°F)* | | | |
|  | **80°F** | **85°F** | **90°F** | **95°F** |  | **80°F** | **85°F** | **90°F** | **95°F** |
| Female, ≥65 yrs | 0.07 | 0.15 | 0.22 | 0.30 | Female, ≥65 yrs | 0.08 | 0.17 | 0.25 | 0.34 |
| Female, <65 yrs | 0.02 | 0.04 | 0.06 | 0.08 | Female, <65 yrs | 0.02 | 0.04 | 0.07 | 0.09 |
| Male, ≥65 yrs | 0.07 | 0.14 | 0.21 | 0.28 | Male, ≥65 yrs | 0.08 | 0.15 | 0.23 | 0.31 |
| Male, <65 yrs | 0.02 | 0.04 | 0.05 | 0.07 | Male, <65 yrs | 0.02 | 0.04 | 0.06 | 0.08 |

Supplemental Figure 3: Confounding illustration using linear association between daily EMS counts and proportion of runs from UHI>0 areas with boxplots representing marginal distributions in (a) residential, low socioeconomic areas, (b) non-residential, low socioeconomic areas, (c) residential, high socioeconomic areas, (d) non-residential, high socioeconomic areas.

Supplemental Figure 4. Daily increase in EMS encounters per 10,000 population, by subgroup, for a weekday in 2021, relative to days with a mean temperature of 75°F in low socioeconomic, residential locations.
